# Quality of care during rural care transitions: a qualitative study on structural conditions

**DOI:** 10.1186/s12912-023-01423-5

**Published:** 2023-08-09

**Authors:** Idun Byberg, Ulla Näppä, Marie Häggström

**Affiliations:** Department of Health Sciences, Mid Sweden University, S-831 25 Östersund, Sundsvall, S-851 70 Sweden

**Keywords:** Continuity of Care, Homecare Services, Nursing, Patient Care Management, Patient discharge, Patient transfer, Rural Health Services, Rural nursing, Transitional care, Quality of Health Care

## Abstract

**Background:**

Registered nurses are critical for the delivery of high-quality healthcare during care transitions from hospital to home. Older co-morbid patients are most vulnerable during these transitions. A growing population of older adults with a higher prevalence of diseases implies increased demands on healthcare and its quality, which is affected by the environment where healthcare is provided. One can draw inferences on the quality of care when classified into structure, process, and outcome. This study explored registered nurses’ perspectives on structural conditions that promote or hinder good quality care during transitions from hospital to home healthcare in rural areas.

**Methods:**

We conducted a reflexive thematic analysis of interviews with 21 registered nurses experienced in care transitions from hospital to home healthcare in a rural area of Sweden. We based the theoretically driven analysis on Donabedian’s definition of structures regarding the quality of care.

**Results:**

The structural conditions were represented by three themes; (I) “Distances and inaccessibility” explains physical matters such as geographical (in)accessibility, bed (un)availability and electronic aids. (II) “Competence of the actors” explains continuity, knowledge and collaboration among the individuals involved. (III) “Levels of organizational governance” explains laws, expectations, values, and agreements regarding care transitions. All themes involved promoting and hindering factors, mutually influencing aspects of the others.

**Conclusions:**

Care actors, educators, managers, and decision-makers need to understand how structures in the physical, social and symbolic environment interactively affect the quality of care during care transitions since understanding this is a prerequisite for improvements. These aspects must be considered to optimize conditions for high-quality care transitions from hospital to rural home healthcare and implemented continuously to improve transitions within the respective organization and inter-organizationally. According to this study, these aspects are critical in a rural context due to structural care quality influencers such as geographical challenges, difficulties in finding competent staff members, development of technical devices, and access to the Internet.

## Background

It is well-known that registered nurses (RNs) are critical to the delivery of high-quality healthcare [1] and have a multifaceted and coordinating role during care transitions from hospital to home healthcare (HHC) [2]. Care transitions refer to a patient’s movement from one location to another for receiving healthcare, such as from hospital to home [3, 4]. Older co-morbid adults are most vulnerable during care transitions [5]. Patients and their relatives may experience transitions from hospital to HHC as an emotional roller coaster, wrought with exhaustion, frustration, and uncertainty [6]. Their satisfaction is linked to care quality [7], although their degree of participation before discharge is not always satisfactory [8, 9]. Life expectancy and disease prevalence among older adults are increasing worldwide [10, 11], and care needs continue to grow [12]. Together with fewer days in the hospital, this poses challenges for RNs in delivering safe, high-quality care [13].

Old age and multiple diagnoses are positively associated with receiving HHC [14, 15]. In Sweden, while the 21 regions provide most healthcare, HHC and homecare services are the municipalities’ responsibility [16]. Most HHC considers the patient’s ordinary residence, but HHC also includes special housing for older adults in permanent and short-term accommodations [17]. The Social Services Act [18] regulates the patients’ right to municipal homecare services. The Health and Medical Services Act [19] and agreements between each region and its municipalities regulate HHC. The agreements are based on the long-term needs and how difficult it is for the patient to access their health center. Inter-organizational collaboration between the regions and the municipalities is thus needed during care transitions from hospital to HHC [20]. RNs have described such inter-organizational collaboration as working in two worlds [21].

Quality of care (QoC) is conceptualized differently across system levels and among professional groups [22, 23]. It usually corresponds to the values ​​and goals of healthcare and the society it is a part of [24]. From a nursing perspective, it includes having a holistic view of the patient [25] and incorporating concepts such as advocacy, caring, empathy, intentionality, respect, and responsibility [26]. Patients and relatives, apart from empathy, add concepts like trust and openness to its purport [27]. The World Health Organization defines care quality as “the degree to which health services for individuals and populations increase the likelihood of desired health outcomes and are consistent with current professional knowledge” [28, 29]. Further improvements require healthcare to be effective, efficient, accessible, patient-centered, equitable, timely and safe [12].

According to Donabedian [30], one can draw inferences on the QoC when classified into “structure”, “process”, and “outcome”. Structure includes material and human resources, and organizational structure. Process means what is done through caregiver and patient actions while giving and receiving care. The outcome is the care’s effects, including patients’ health status and knowledge. These three concepts interact since structural improvements affect the processes, leading to patient outcomes [30]. Research focusing on these can increase understanding of care quality [24], such as by drawing inferences about the quality-of-care transitions when using readmission rates as an indicator of outcomes [31, 32]. Studying settings where the care processes occur can be beneficial, as proper settings and instruments are prerequisites of good care [24]. As the path to patient safety runs through the actors delivering care, healthcare organizations should be designed to support their actors in delivering high-quality healthcare [33].

The connection between the environment, nursing and patient health is well-known [34]. The environment concept constitutes one of four critical typologies within the metaparadigm of nursing [35], and is essential in developing nursing knowledge, as it forms the context of nursing practice [36] and impacts the QoC [37–39]. The concept incorporates the physical environment, which is matter-based, the social environment, referring to individuals with whom the person interacts and the symbolic environment, meaning values, laws, and expectations [40]. It is thus closely linked to Donabedian’s definition of structures contributing to the QoC, including material, human and organizational aspects [30].

Despite goals for care on equal terms [19], there are still geographical inequalities regarding HHC. Rural areas often lack infrastructure and transport options as found in urban areas. Further, the residents are often older and have a greater need for HHC [12], while the number of people employable in HHC is limited [12, 41].

Research has shown the structural conditions that affect the ability to deliver good quality care during care transitions from hospital to home. These are material resources like access to electronic aids [42, 43] and bed availability, human resources such as the patient’s health status [44], and staffing issues [43, 45], and organizational structures such as national expectations [46]. However, despite environmental inequities between urban and rural residents, to our knowledge, no previous studies have explored these aspects from a nursing perspective, focusing on rurality. Increased knowledge of the structures affecting the QoC could contribute to continuous improvement. Therefore, the research question of interest to this study was: what prerequisites do RNs consider themselves to have to provide good quality care transitions from hospital care to home healthcare in rural areas? The aim of this study was to explore RNs’ perspectives on structural conditions that promote or hinder good quality care during transitions from hospital to HHC in rural areas.

## Methods

### Design

This study is a supplementary qualitative analysis [47] of interview data from a previous study [48]. Both studies provide knowledge of RNs’ perceptions of QoC in transitions from hospital to HHC in rural areas, while inferences on the QoC can be drawn when classified into “structure”, “process”, and “outcome” [30]. The primary study aimed to provide a deeper understanding of what RNs’ perceive as the main concerns in care transitions from hospital care to HHC in rural areas, and how they handle these concerns, reflecting their perspective on the process. This second study aimed to explore RNs’ perspectives on structural conditions that promote or hinder good quality care during transitions from hospital to HHC in rural areas. This theory-driven analysis, guided by Donabedian’s clarification of structure (30), was conducted using reflexive thematic analysis (RTA), as described by Braun and Clarke [49]. Social constructivism underpins this study, meaning knowledge is relative to time and place, worldviews change over time, and the idea of multiple, subjective realities is central to this inquiry. The researcher’s objectivity ​​is thereby impossible, which entails demands for reflexivity and a willingness to examine one’s pre-understanding not to diverge much from the data during the inevitably subjective interpretation [50], which is central to RTA [49] and described in the analysis section.

### Setting and participants

We conducted this study in northern Sweden. Participants worked either at a county hospital or HHC in one of the municipalities whose residents’ secondary care belonged to the same county hospital. In all municipalities, the majority population had at least 45 min and an average of 90 min of travel time to reach an area with a population of at least 50 000 [51]. Sampling was initially purposive and later developed into theoretical by adding new participants and questions following the grounded theory method used in the primary study [52, 53].

Inclusion criteria were RNs experienced in care transitions from hospital to HHC, working in either. The number of potential participants who declined participation is unknown since the RNs received a videotaped and written offer to participate through their managers, who had previously approved the research. Twenty-one RNs participated; their demographics are displayed in Table 1. All had a bachelor’s degree in nursing, and some had undergone specialist training in district nursing, eldercare, oncology, or medicine, thereby acquiring a master’s degree.

**Table 1:** Participants’ Demographics

Occupation	Years of service as RN	Specialist trained RN	Age	Gender		Maximum distance from office to patient’s home
				Female	Male	
Hospital (n = 8)	2–25 y (md 9,75 y)	1	26–50 y (md 38 y)	7	1	250 km
Home healthcare (n = 13)	6–30 y (md 18 y)	13	31–64 y (md 51 y)	13	0	150 km

### Data collection

The first author carried out one individual interview with each participant in August-October 2021, at a time and place of the participants’ choice, either face-to-face (n = 10), through Zoom or Teams (n = 9), or by phone (n = 2). By offering the participants to choose how the interview would take place, we could not observe body language and gesticulation in some interviews. However, no difference in the information richness in the interviews was identified, whereby the approach may be considered a strength because it can optimize the participants’ comfort and strengthen their empowerment in the relationship with the interviewer [54]. The interviews lasted between 63 and 149 min (md = 83 min) and were audio-recorded and transcribed verbatim. An interview guide, jointly created by us authors, enabled exploring the participants’ experiences with the research topic. Before interviewing the participants, two test interviews not included in this study were conducted with RNs to evaluate the interview guide. The initial question was, “How do care transitions from hospital care to homecare take place?” followed by questions like, “Can you please tell me about an occasion when a care transition went well?”. Questions like “Can you please elaborate on that?” were asked to gain further knowledge of the participants’ perspectives. We developed the interview guide parallel to interviewing in a quest for theoretical saturation of the primary study. We thus added questions like “Who is responsible for the patient during transport from hospital to home?” and “What do you do as a nurse when you discover that a transition has not gone well?”. The participants were welcome to speak freely without interruption.

The perceived re-usability of the data must be considered when planning a secondary analysis [47, 55]. A potential problem in making qualitative secondary analysis based on data from a primarily grounded theory study is that data collection is an iterative process shaped by ongoing analysis, which risks covering some topics in more depth than others [47]. The inductive interviewing generated very rich material, which, in addition to the RNs’ actions answering the primary study’s purpose, also revealed affecting structures. Open-ended questions tend to cover more questions than those leading to the grounded theory-related core category, whereby a secondary analysisis is a responsible way to preserve existing data in a position to answer previously unanswered questions [47]. Both the primary study and this study are thus based on the same dataset generated by the same open-ended interview questions about the RNs’ perspectives on QoC. Another potential problem in making qualitative secondary analysis is the fit between the study’s aim and the data [47]. Since studies aimed to explore RNs’ perceptions of QoC in transitions from hospital to HHC in rural areas, focusing on different aspects of QoC, the data fit the aim.

Besides the audio recordings, notes were taken during the interviews to facilitate reverting to previously discussed aspects and asking more about them. All participants declined the offer to correct the transcripts, which is a way to member-check them [56]. The transcripts were compared with the audio recordings by the first author to ensure their validity. Each participant was assigned a code number (e.g. P1, P2, P3) to guarantee confidentiality.

### Analysis

We based the qualitative secondary deductive analysis on Braun and Clarke’s six phases of RTA [49, 57], which requires constant reflections on one’s pre-understandings, critically examining their impact on the interpretations made. Before analysis, the first author raised awareness using a reflexive writing exercise designed by Braun and Clarke [49].

In **Phase 1**, the first author read and re-read the transcribed interviews to familiarize herself with the data concerning this study’s aim, guided by Donabedian’s description of structure (30). The Swedish-language transcriptions were then imported into the software Nvivo for manual analysis. Considering the large amount of data, the software eased the overview. Each of the six analysis phases had its separate file in Nvivo, facilitating the iterative process since the previous analysis phase remained untouched, facilitating return when required.

In **Phase 2**, every text segment of potential interest received codes by profession or structural area, shortly labelled, e.g., “managers”, “agreements”, etc. The codes were within the theoretical domains of material resources, human resources, and organizational structures. Pre-understandings related to interpretations of data were criticized through reflexive memos, shared, and discussed with the co-authors to ensure that interpretation did not diverge much from the data. Most were written in Nvivo, and some linked to the codes that raised those analytical considerations to keep analysis and reflexive memos close, enabling their critical review in later analysis phases. The reflexive analytical thoughts included illustrative figures to ascertain how different structural aspects could conceivably fit together and affect the QoC.

In **Phase 3**, the first author reviewed each code and re-named it with a more precise explanatory label. When codes contained similar structures but contrasting meanings, they were combined in a separate Nvivo file to further recode for analytical clarification. This opened alternative analytical possibilities, enabling the collapsing of codes into unexpected clusters. Some text segments of potential interest, coded in the previous phase, were sorted out through discussions between the authors. In this phase, the illustrative figures helped interpret how parts of the different structures influenced each other despite belonging to different theoretical domains.

In **Phase 4**, the authors created new interweaving concepts and preliminary codes clustered to build preliminary themes, whereby some codes moved between initial domains and others were excluded. Each preliminary theme was assigned a label describing its content. Codes were compared with others within the same cluster to determine whether the ongoing sorting was reasonable, considering the study’s aim and data. Some codes could unexpectedly come together in the same preliminary theme.

In **Phase 5**, minor sorting adjustments were made by moving codes between themes. The first author began a draft summary in Swedish for each preliminary theme to further define and clarify each content. While summarising each theme, it became apparent which codes did not belong and which did better in another theme and needed to be re-sorted. The first author then translated the draft from Swedish to English, and the content and concepts of each theme were refined. We regressed to codes and text segments in Nvivo to ensure the analytical interpretations in the draft were sound according to the data.

**Fig. 1:**
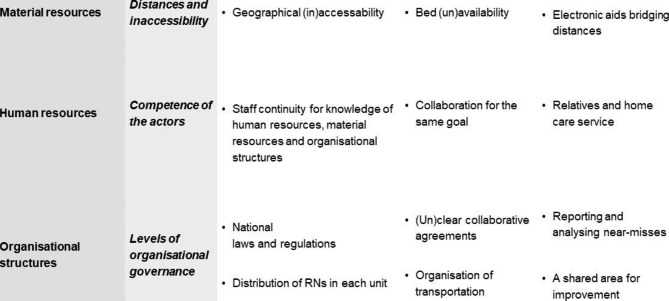
An example of clustered coding within the structural domains of quality of care in transition from hospital to rural home healthcare

In **Phase 6**, we mutually excluded some additional codes, as in an English written format, they no longer seemed to answer the study’s aim or fit the theme. Thus, a new level of critical reflection occurred in this phase. We used analytical illustrations throughout the previous phases to elaborate on connections within and between the themes. This phase included telling the analytical process and combining the results with previous research in the discussion. The figures presented in this study, based on illustrations made throughout the analysis phases, were completed during this phase, and guided the writing of the final draft. See Fig. 1 for an example of the clustered coding.

### Ethical considerations

When re-using qualitative data in secondary analysis, the ethical guidelines apply equally to data treatment in primary research [47]. Values in The ICN Code of Ethics for Nurses [58] underpinned research practice. Participants received oral and written information about the purpose of the research, the voluntariness of participation and they were ensured confidentiality. Participants’ integrity and empowerment were highly valued, whereby they were offered to decide the time and place for the interview. Each participant was pseudonymised with a number (P1-P21) to ensure confidentiality. We handled the data following the General Data Protection Regulation [59], and the interview transcripts were accessible only to authorised researchers and used solely for academic purposes. The Consolidated Criteria for Reporting Qualitative Studies (COREQ) was used for study reporting [60].

## Results

**Fig. 2:**
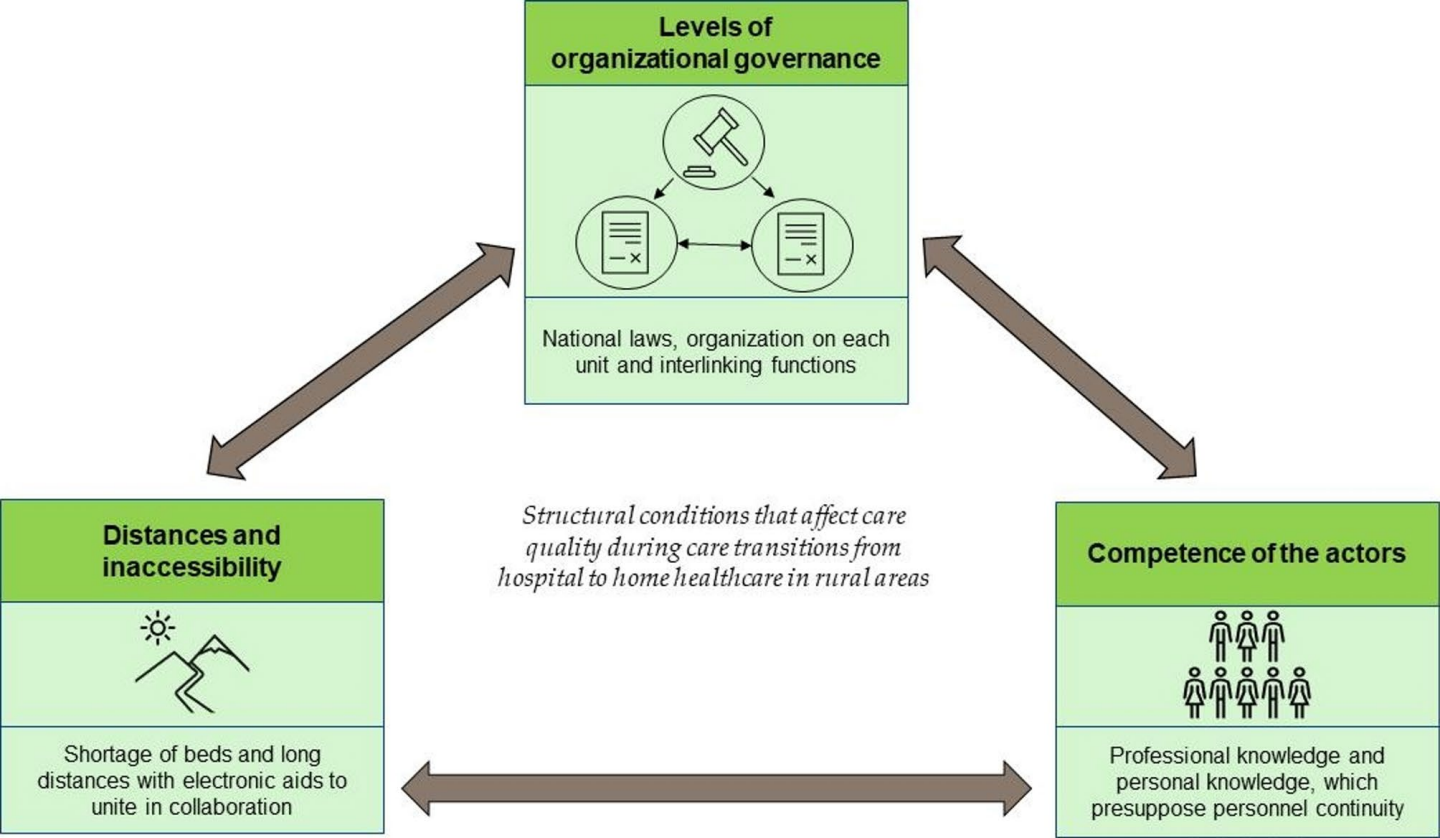
An illustration of the structural conditions that are involved one-to-one and affect the quality of care in transitions from hospital to home healthcare in rural areas

The three themes *“Distances and inaccessibility”, “Competence of the actors”*, and *“Levels of organizational governance”* represent RNs’ perspectives on structural conditions that promote or hinder good quality care transitions from hospital to homecare in rural areas and are illustrated in Fig. 2.

### Distances and inaccessibility

The care transitions occurred in an environment of long geographical distances and inaccessibility, including unavailability of beds and resources such as electronics to bridge the distances. The homecare nurses had a long way to the hospital, patient’s homes, pharmacies and assistive equipment centers. The rural roads were poorly maintained, threatening the patient’s well-being during the transit from the hospital to home. The homecare nurse’s distance to the patients’ homes and the number of homecare nurses in each geographical area affected the required staff resources in the homecare social service to deliver healthcare at home. It would have been ideal for physicians to consider the distance’s impact on the need for staff resources when prescribing drugs before discharge, in case some alternatives required less frequent administration.

*“When the patient is prescribed a treatment, they have the right to receive it, regardless of whether they live in a rural or urban area, it shouldn’t matter. However, if there ***is ***another treatment option, then you can consider it.”* (P8, homecare nurse)

Long distances to pharmacies and assistive equipment centers meant long delivery times, while the homecare nurses had limited supplies in their stores. This inaccessibility emphasised the benefit of the regional level of organizational governance, having an inter-organizational agreement stating the hospital’s obligation to send the patient medicines and assistive equipment upon discharge.

*“Our agreement states they shall send medicine for five days. So maybe they send a dosette box from the hospital for five days. Then I have time to get it [the medicine] from the pharmacy.”* (P1, homecare nurse)

Further inaccessibility concerned bed shortages. The high pressure on the few beds in the hospital could cause the admission of the patients in a different ward than the specialised one appropriate to the patient’s condition, or fully treated patients having to change wards while awaiting discharge. Caring for the patient in the correct ward based on the current reason for admission and with as few changes of wards as possible was associated with good QoC. The shortage of hospital beds created a need for the municipalities’ short-term accommodations to enable the flow of the care chain. However, short-term accommodations were also in short supply.

*“First of all, I think the municipalities must have their short-term beds … not letting patients who have been fully treated stay in an emergency hospital, that’s number one.”* (P11, hospital nurse)

Short-term accommodation was a valuable place for a stopover from the hospital to HHC when discharging the patient to a rural area, where help was far away if suddenly required. Some care needs were impossible to be met since the long distances required considerable staff resources, based on increased transit time for home visits.

*“If intermittent catheterization is really necessary, then the patient cannot come home. Then we need to discuss other alternatives. Short-term accommodation, catheter or...”* (P10, homecare nurse)

Short-term accommodation enabled discharge even when care needs were too great to be met in the patient’s home. A stopover there was considered safe, based on the recovery time the patient required before going home.

*“It [decision on discharge] can go fast, as the patient is considered to be in stable performance status based on the reason for admission so that he can be discharged. Nevertheless, he may still need recovery to be able to return home. Then there must be these assets, short-term beds and so on.”* (P20, hospital nurse)

Electronic aids enabled good quality care during care transitions, where the actors were separated by distance. A shared digital e-message system was a promoting factor in bridging the geographical distances between the actors. This system made everyone’s functions easier, while everyone doing their part was also a prerequisite for relying on what was written. It was perceived as relieving the RNs somewhat in their coordinating role.

*“That is the idea with this e-message system; everyone should do their part. It is not one person who should arrange everything–as the nurses’ role usually has been.”* (P15, homecare nurse)

The RNs felt secure having the agreements in print. They believed it reduced the need for telephone contact, which was positive since the telephone network could be unreliable in rural areas. The digital e-message system gave all actors access to messages concerning the patient’s Activities in Daily Life (ADL) status in the hospital and which interventions were granted by the municipality.

*“When you open that communication channel to the municipality, social workers and municipal nurses can read. They get access to certain texts relevant to the admission. It is almost like a chat program.”* (P9, hospital nurse)

A negative aspect of the digital e-message system was its fragility in the case of electronic clutter. Additionally, the record access was unilateral. The hospital nurses lacked access to the municipal records, which they thought would facilitate assessments of new symptoms of the patients who had HHC before hospitalisation. Further, digital aids were not used to the extent possible. Homecare nurses wished they could have digital care planning meetings through video calls, as they promoted patient participation.

*“If you were connected like we are now [during the interview via Zoom] so you can look at each other, then you could plan. The patient may be sitting with the care planning nurse, a relative and the social worker. We could plan how we think and what we shall do. With a webcam, you get a dialogue ... so you can see each other... That is our wish, among other things. […] To me, it would feel like the patient is more involved.”* (P17, homecare nurse)

### Competence of the actors

Insufficient knowledge was perceived to impact the QoC significantly. Sufficient knowledge meant knowing what one had to deal with and feeling secure in various aspects of nursing. The RNs noticed insufficient knowledge in others. While most hospital nurses thought they had insufficient knowledge about inter-organizational agreements, homecare nurses said they noticed this during care transitions. Correspondingly, hospital nurses perceived some homecare nurses’ uncertainty about specific aspects of nursing, which the latter also confirmed. Insufficient knowledge involved confidence in handling the digital e-message system, which RNs in both organizations considered lacking. For several RNs, it was unclear who was responsible for the patient during physical transport from the hospital to home in case something happened.

*“I think as long as the patient is not at home, there is no one who... it feels like no one is responsible if something happens. […] I feel like no one is in charge during the transition, leaving the ward and heading home. There is none responsible, or we are poorly informed about who is responsible.”* (P21, hospital nurse)

The competence of the actors in care transitions included continuity, a knowledge prerequisite. Knowledge included professional knowledge, what to relate to, and personal knowledge of the patient and other actors. This study showed that as many as 24 actors could be involved in a care transition. Besides the patient, there were formal and informal caregivers and staff within other inter-organizationally linked activities, such as taxi drivers and the assistive equipment center staff. The importance of each actor varied based on individual patient-related factors such as health status and help need and whether the care transition was considered from the perspective of hospital nurses or homecare nurses. The care chain functioned like a “staircase” (P5, homecare nurse), where one actors action depended on the previous actors.

*“There are so many other big pieces in a transition. There are so many people involved.”* (P6, homecare nurse)

Knowledge of what to relate to presupposed knowledge of organizational structures, such as laws and agreements, and material resources, such as environmental conditions. Everyone having the same goal and attitude was essential to the QoC. This was evident regarding transitions of patients with palliative care needs, where everyone often did their utmost to reach the same goal.

*“Well, when it is palliative care, she [the patient] is going home as soon as possible. Then it is as everything just flows as if everyone has the same attitude and the same goal.”* (P4, homecare nurse)

Insufficient knowledge of what each actor had to relate to decrease the understanding and trust in other actors’ actions. Several homecare nurses felt that hospital nurses lacked knowledge of their conditions. Hospital nurses too confirmed that they experienced frustration when they did not understand why homecare nurses did what they did.

*“It is important; what role do you have in this, what role do I have, and what role does the actor have? It contributes to why it does not work so well that we sometimes do not have a clue. I don’t know what everyone is doing, so it’s necessary [to increase the understanding]. We should have a better understanding of each other’s function and each other’s workload.”* (P4, hospital nurse)

The lack of understanding was assumed to be due to more homecare nurses having previous experience in hospital care than the other way around. At the same time, homecare nurses also did not understand hospital nurses’ actions. Lack of knowledge of each other’s conditions created frustration that could affect cooperation in future care transitions.

*“…what can I say, you get a little bad mood between inpatient care and rural, municipal care.”* (P20, hospital nurse)

Adequate personal knowledge included knowledge of the patient. Hospital nurses thought they had poorer knowledge of the patient than the assistant nurses, who saw the patient the most. The homecare nurses thought they knew the patient well because they had HHC before the hospital stay. Knowledge of the patient included their knowledge of themselves, and their help needs. Cognition influenced patient participation, whereby cognitive impairment in the patient increased the risk of worse care transitions, as patient participation was associated with good care quality.

Relatives were a resource of knowledge about the patient. They could help identify the patient’s needs, and act as a link between the patient, the home, and the formal caregivers. A prerequisite, however, was that they were sufficiently informed and understood how newly acquired conditions could affect the patient’s changed conditions after discharge. Care transitions were considered more fragile and riskier when close relatives were missing. Close relatives could also include neighbors. Hospital nurses with long experience in rural areas assumed that social safety nets differed from urban areas. One of them expressed it thus:

*“...when it comes to sparsely populated rural areas, as there can be a long distance to the closest neighbor, they still keep an eye on each other, like a small security team of their own.” (*P20, hospital nurse)

Sufficient homecare service staffing was required for HHC, and hence discharge. The staff needed to be experienced and knowledgeable to meet the patient’s needs. It concerned professional knowledge as an assistant nurse and patient knowledge, where continuity was important. In a small community, there was usually a good knowledge of the residents. Where healthcare facilities were close, the patient could sometimes have the same staff in HHC as during a stay in short-term accommodation, which was positive in care transitions from the hospital to home through short-term accommodation. Staff continuity in a small rural community contributed to good and close collaboration between the homecare nurses and the general practitioner responsible for primary care, based on mutual knowledge of the patient and the conditions for rural HHC. Homecare nurses perceived an increased risk of re-enrollment of the patient where the general practitioner was an agency doctor since they lacked knowledge of the patient, the environmental conditions, and inter-organizational agreements.

*“In the end, it is the doctor who decides. If the doctor has met this patient, it has an effect because he has a picture of his own. However, if he has not, he might think, `Well, send him in [to the hospital]’”* (P14, homecare nurse)

Hospital nurses too connected knowledge to staffing continuity. They felt they had better opportunities to assess care needs when caring for patients whose diseases they were familiar with. Therefore, the patient needed to be in the correct department, based on the reason for admission. Many new assistant nurses without experience made cooperation between them and the hospital nurses difficult. If short-term hired agency doctors arrived on a Monday without knowing the patients, it could delay discharge. Further, the continuity of unit managers was essential to good quality care. Improved working methods in the ward were considered difficult to implement if the unit managers did not remain in service for long.

*“Lack of routines. We have changed managers several times. It worked great when we had a manager who pushed things forward and had control of the situation, as it was in the old times. Then I thought it was great fun to work.“* (P3, hospital nurse)

### Levels of organizational governance

Organizational governance occurred nationally and locally, within each unit and inter-organizationally. The national governance concerned legislation and overall regulations regarding the division of responsibilities between the region and the municipality. One statutory aspect was that the municipality became liable to pay the region for each day the patient remained in the hospital as ready for discharge. The RNs felt that actors within the two organizations fighting for different financial interests and the issue of economic responsibility being prioritised over patients’ well-being, was frustrating. Some felt it might be better if regional and municipal care belonged to the same organization.

*“So, it is actually about money. It is about who is responsible for costs and everything. [If it were] The same healthcare organization, one and the same payer, oh, how good it would be then.“* (P17, homecare nurse)

A further strongly influencing aspect was that different actors worked under different laws, entailing different rights. The social worker who operated under the Social Services Act had nothing to do with the Health and Medical Care Act and was, therefore, had access not to the hospital’s patient records, but only to messages in the digital e-message system, such as ADL status. It was a common belief that the social worker did not get as good a picture of the patient as the homecare nurses, while the homecare nurses lacked decision-making rights under the Social Services Act. Consequently, RNs feared that the homecare services’ interventions granted based on the Social Services Act would be insufficient vis-a-vis the patient’s needs.

*“So, what I can miss, is that the social workers have a different picture from me because they do not have access to the whole patient record. We have one patient now who is unfamiliar. The social worker called the patient. However, she [the social worker] does not know the memory test was not that good.“* (P12, homecare nurse)

Another strongly impacting statutory aspect was the patient’s rights regarding autonomy and confidentiality. The patient’s own will weighed the most, regardless of the degree of insight into the disease and how it could affect abilities for self-care. According to the RNs, this risked bad patient outcomes when the patient objected to receiving care at home after discharge.

*“The patient may look very entitled to home healthcare and a lot of Social Service interventions. However, they also may say no to a lot. The social worker goes for the self-determination autonomy principle. This ethical principle is that the patient should be allowed to decide about his own care.“* (P5, homecare nurse)

Further, patients decided whether relatives were to participate in the care planning due to their right to confidentiality. Another aspect of confidentiality was that homecare nurses were not allowed to enter the conversation in the digital e-message system before it was clear they would be involved in the patient’s care after discharge. They felt this hindered the possibilities for proactive planning associated with good quality care.

*“When I read and follow, I can feel that this is coming up; it is probably someone I will visit at home. However, I can’t ask if this patient needs home healthcare interventions because it is not my responsibility* [to ask].*“* (P2, homecare nurse)

An additional organizational influencing structure was local within each unit, which involved the distribution of RNs. Over the years, the distribution in the hospital had developed from hospital nurses being responsible for approximately 4–5 patients to 7–10 patients. Parallelly, patients were perceived to be more medically complicated than before due to more conditions becoming treatable. The fact that hospital nurses needed to focus on several patients simultaneously led to low priority being accorded to non-urgent tasks such as care planning, and the number of patients per RN was not always perceived to be safe. In HHC, the problem with homecare nurse distribution mainly concerned the number distributed over the day. Homecare nurses worked during the day, with a couple of on-call homecare nurses at night, who were said to have inadequate time to receive patients from the hospital during the evening and night. The distribution of homecare nurses entailed the reduced possibility of discharging the patient during other parts of the day than the forenoon. However, based on the sought-after care flow, the goal was to discharge patients whenever they had completed treatment.

*“We work from a quarter past seven to half past five, and then there are night-shift nurses who don’t have the opportunity to receive patients. We have two [night-shift nurses], they cover the whole municipality, and it is a very long distance. So, it must be fixed within a reasonable time of the day.“* (P5, homecare nurse)

Another local organizational structure affecting the possibility of providing good quality care was the routines and guidelines at each unit. Hospital nurses felt a lack of routine for raising care planning matters on rounds. Organizational governance also involves guidelines for inter-organizational collaboration. An inter-organizational agreement described a specific workflow regarding what each organization did during care planning before discharge. However, as errors continued, this did not eliminate the risk of something being left behind at the hospital. The agreement regulated who would be entitled to HHC based on specific criteria. The homecare nurses perceived the criteria as being based on the homecare services granted by the social worker according to the Social Services Act. The social workers’ decision was based on ADL status, as shown in the digital care planning system. It was often the assistant nurses in the hospital who assessed the ADL, on which social workers based their decision about homecare services, guiding the homecare nurses’ decisions about HHC. Homecare nurses found this workflow questionable concerning care quality since they thought some people could end up “between the seats” (P14, homecare nurse) if they strictly followed the agreement.

*“Many people may need interventions based on the Health and Medical Care Act even if they do not meet the requirements to receive it.“* (P6, homecare nurse)

The criteria for HHC were perceived to be unclear, leading to ambiguities regarding the responsibility for the patient’s care after discharge. They were not always perceived to correspond to the actual needs for HHC that nurses thought they identified. In the event of ambiguities, some HHC managers called for adherence to the agreement, while others called for a departure therefrom. In cases where the criteria were unmet, the region could transfer only the patient’s medication management responsibility to HHC or purchase single nursing services. However, such sharing of responsibility for different parts of care was not perceived to be in the patient’s best interests. It risked a fragmented perspective of the patient’s status and needs, rather than an overall picture.

*“If they need help from the municipality, then many service purchases must be made. That complicates it more. It becomes more cumbersome and doesn’t feel like a good solution for the patient.“* (P20, hospital nurse)

The organization of transport between the hospital and the municipalities was a further influencing structure. Firstly, there was a single assistive equipment center with associated cars for weekly municipality deliveries. Secondly, there was an agreement with sick-leave taxis for departures from the hospital at fixed times during the day. These two transport organizations enabled, for example, the forwarding of forgotten items after discharge.

*“That forgotten medicine, I think it had to go with the next taxi. It [problems] can usually be solved.“* (P12, homecare nurse)

On the other hand, homecare nurses found that the journeys home in the sick-leave taxis were often difficult for the patients. The fixed times for departure in the afternoons were not optimal because of the long travel times. Further, there was usually carpooling, which was exhausting for some old, sick people since it meant extended travel times. The carpooling also made it difficult to calculate the time of arrival to meet the patient at home. Not all patients were entitled to sick travel taxis but had to arrange the journey home independently.

“There may not even be communication links at the far end of the sparsely populated rural areas. You automatically think that someone should be able to take the bus. The patients may not have ridden a bus in 40 years. Imagine that *** [patients name], 82 years old ... walks to the bus station and takes the bus home to *** [rural place], that’s … Yes, it’s gut-wrenching.“ (P20, hospital nurse)

The management of near-miss incident reports was another structural inter-organizational aspect of value for the QoC. RNs regarded it as an essential part of the improvement work. HHC managers and hospital nurses called for the homecare nurses to report near-miss incidents. Regular coordination meetings were held between the regional and municipality care managers to identify shortcomings in cooperation. However, the RNs considered sparing the manager’s feedback on the measures the near-miss incident reports led to. The feedback was communicated at workplace meetings at the hospital but was not compulsory in all wards. In HHC, feedback similarly took place at collaboration meetings, but feedback could also be completely lacking.

*“What their and our managers say is that we have to bombard with near-miss incident reports so that the regional management will understand that they are wrong.“* (P10, homecare nurse)

## Discussion

This study explored RNs’ perspectives on structural conditions that promote or hinder good quality care during transitions from hospital to HHC in rural areas. Donabedian’s model for care quality, with a focus on structures, was the theoretical springboard for the reflexive thematic analysis. Through the analysis, we created three themes, viz. *distances and inaccessibility, competence of the actors*, and *levels of organizational governance*, all explaining factors both promoting and hindering good quality care. The result shows how different factors within and between these themes influence and are influenced by other factors. By presenting the factors regarding material and human and organizational factors, rather than focusing on either, this study offers an overall understanding of the interacting conditions affecting the care transition in a rural context. We demonstrate the theoretical usability of Donabedian’s model of care quality, where he brought three domains within the so-called “structures”. This study can provide a deeper understanding of how these structures affect the QoC in the rural context, which we believe is required for optimisations.

The conditions presented in *distances and inaccessibility*, such as distances between caregivers, bed shortages, and electronic aids as affecting factors, agree well with previous studies’ findings [42–44]. However, few or no previous studies considered these factors, focusing on rural areas. This study is thus a novel contribution, as it explains how, for example, the distance between the hospital and the home can affect the time of discharge and how bed shortages in hospitals and short-term accommodation affect each other in the care chain.

In the theme *competence of the actors*, continuity, knowledge and having the same goal are presented as essential factors in the theoretical domain of human resources. Previous research [61] shows that skill shortages in rural areas are problematic. Rural areas face challenges recruiting RNs due to lack of availability because RNs comfortable working in rural areas are hard to replace [62]. These problems may thus directly affect the quality of the care transitions negatively. This study highlights the importance of continuity as a prerequisite for knowledge when working with care transitions. Besides recruitment, it highlights the importance of retaining staff over extended periods to ensure continuity.

A uniform care plan is associated with high-quality care transitions [63]. However, previous research [64] also shows a lack of understanding of each other’s work situation, creating distrust, which may not be unique to rural areas. However, this study describes how this mistrust can manifest in care transitions when RNs in hospitals and HHC, as in Sweden, work within two different organizations. The different organizational affiliations entail using different record systems, and different actors having access to different parts of the hospital patient record, which is a further complicating factor, as it can give a fragmented perspective of patients and their care needs, which is vital for policymakers to consider. Further, the importance of the actors’ knowledge of the rural context is also emergent from a nursing perspective, as shown in a previous study of care transitions from hospital to HHC in rural areas [65].

Perhaps the most overall affecting theme was *levels of organizational governance*, and it explains influencing organizing from national to local levels. The theme features, for example, the importance of reporting near-misses and having a shared area for improvements. The safety culture within each organization was influential, and the tendency to report near-misses and the value of doing so were significant. Patient safety culture may be considered an integral part of the QoC, whereby we interpret it as an essential organizational aspect during care transitions. The feedback to the RNs involved in the near-misses differed, while previous research [27] has shown that dialogue and feedback are considered necessary. The importance of handling near-misses and the shared area for improvement is probably not unique to remote areas or the Swedish context and is probably transferrable to the point that nursing managers should constantly recollect that leadership approaches bear significant responsibility for the patient safety culture and error management [66].

This study exemplifies how the knowledge and participation of patients and relatives are valuable resources in care transitions, which also require their knowledge. It may clarify the importance of RNs, like other healthcare personnel, ensuring that their knowledge needs are met and they are well prepared for the care transition [63]. This presupposes the RNs’ knowledge of care transitions, highlighting the importance of professional teaching institutions, including care transitions and improvements in their education. Further, as this study shows, there must be sufficient conditions in all structural domains to conduct care transitions of good quality. Thus, policymakers must consider these complex, context-specific aspects when designing guidelines [63]. All actors involved in the structural conditions of care transitions must understand their interactions since adjustments in one may affect the others. The physical, social, and symbolic care environment plays an essential role.

To the best of our ability, we quoted the participants to strengthen the study’s credibility and described the context for facilitated transferability. Joint discussions to maintain alertness to our subjectivity strengthened the study’s confirmability and trustworthiness [50]. By using self-collected data in this study, we eliminated the potential problem with secondary analysis concerning the researcher’s knowledge of the context of data collection by involvement [47]. This secondary analysis was carried out relatively close to the data collection, whereby the potential issue of researchers “not having `been there’ - for a while " [47] was eliminated.

In qualitative research, where researchers are the instrument for data collection and interpretive analysis, the researcher must critically reflect on potential sources of error [50]. Therefore, we chose to write in the first person instead of the academic tradition of the third person, as it clarifies our active roles and demonstrates our awareness of our perspectives as part of reflexivity [49, 50]. We have carefully used the 15-point checklist for good RTA during conduct to ensure the quality of this study [49].

This study explains the importance of access to short-term accommodations for facilitated care flow through the entire care chain, not least in rural areas with long distances and limited personnel resources. Expectations of an increasing need for care [10–12] suggest a pressing need for intermediate departments to ensure good quality care in care transitions. This study shows that patients can be considered too ill to move directly from hospital to home. Therefore, it would be interesting for researchers to examine how access to short-term places or intermediate departments affects the QoC in care transitions in rural areas. This study also explains the importance of the competence of the actors, which highlights the need for implementing care transitions within education for actors involved in care transitions, such as nurses and social workers. This study shows that as many as 24 actors could be involved in a care transition, which exemplifies one of the organizational challenges of care transitions. Not least related to the increasing multi-diagnosed older population needing advanced medical care at home [10–12], at most risk during care transitions [5]. It highlights the importance of continuous improvement work regarding the organization of transitional care to avoid a large group of actors and associated near-misses and incidents. The results show that the RNs had insufficient knowledge of each other’s conditions, which led to a lack of understanding of each other’s actions. Clarifying each involved actor’s work environmental conditions may seem essential for successful collaborative teamwork, whereby the importance of surrounding structures should be highlighted in the professional education of actors involved in care transitions.

Further, educational interventions regarding care transitions may help investigate the effects of competence more closely. Perhaps educational specialization focused on the care chain is required to orientate in today’s multi-organizational healthcare system. Health systems and healthcare providers must consider the contextual variation between different geographic areas [3]. While care needs are increasing [12], the demands on offering advanced HHC can be expected to increase, which means more care transitions and an increased need for knowledge in this field. Adding different perspectives from the many actors involved might be one way to increase knowledge. It would be interesting to have future studies deal with the perspective of patients and relatives on their opportunities and abilities to influence both their care transitions and the improvement work regarding care transitions.

## Conclusions

This study is a novel contribution highlighting RNs’ perspectives on structural conditions regarding material, human and organizational resources that affect the QoC in transitions from hospital to HHC in rural areas. The aspects presented in the three themes, *distances and inaccessibility, competence of the actors*, and *levels of organizational governance*, explain conditions that mutually influence aspects of the others of importance to consider in the continual improvement work and to overcome challenges encountered during these care transitions. RNs, managers, educators, and policymakers must understand and consider how they interactively affect the QoC, to optimize the conditions for high-quality care transitions in rural contexts. Each organization should develop a holistic view of the physical, social and symbolic environment, simultaneously affecting the QoC, and concerning inter-organizational collaborations, to avoid possible mistakes or safety risks during the hospital to rural HHC transitions. According to this study, these aspects are critical in a rural context due to structural care quality influencers such as geographical challenges, difficulties in finding competent staff members, development of technical devices, and access to the Internet.

## Data Availability

The datasets used and analysed during the current study are available from the corresponding author upon reasonable request.

## Abbreviations

HHC: Home healthcare
RN: Registered nurse
RTA: Reflexive Thematic Analysis
QoC: Quality of Care
