# Correction: Quality of care during rural care transitions: a qualitative study on structural conditions

**DOI:** 10.1186/s12912-026-04742-5

**Published:** 2026-07-14

**Authors:** Idun Byberg, Ulla Näppä, Marie Häggström

**Affiliations:** Department of Health Sciences, Mid Sweden University, S-831 25 Östersund, Sundsvall, S-851 70 Sweden

**BMC Nursing (2023) 22:262**

10.1186/s12912-023-01423-5

In the original publication, the following corrections are incorporated:

In this article the corresponding author’s name was Idun Winqvist and now the author’s name has been changed to Idun Byberg.

In this article the e-mail address of the corresponding author was given as idun.winqvist@miun.se where it is updated as idun.byberg@miun.se.

In this article the corresponding author’s initials in Acknowledgements, Author contributions, etc. was I.W where it has been changed to I.B

The original article has been corrected.

